# Development and Sensory Assessment of Ready-to-Eat Breakfast Cereal

**DOI:** 10.1155/2022/4566482

**Published:** 2022-08-12

**Authors:** Theresa Gyamfuwah Frimpong, Faustina Dufie Wireko-Manu, Ibok Oduro

**Affiliations:** Kwame Nkrumah University of Science and Technology, Ghana

## Abstract

There is a gradual change in the eating trend of Ghanaians. People now prefer convenient semiprocessed foods as breakfast meals to raw ones. These breakfast meals make use of cereals and grains, which often suffer postharvest losses. Thus, this study was aimed at adding value to these food crops by producing a nutritious convenient breakfast meal in the form of flakes using yellow maize and coconut as main food components. Five different formulations of percentages, maize against coconut (80/20, 77.5/22.5, 75/25, 72.5/27.5, and 70/30), were developed using the Design-Expert's D-optimal design to produce the breakfast meals through drum drying and the products assessed for acceptability by consumer panel. Panellists rated the produced cereal high in overall acceptability during the sensory evaluation. The overall acceptability decreased with decreasing coconut percentage in the cereal product. The 70/30 formulation was most preferred by panellist. The protein, fat, ash, fibre, carbohydrate, and energy contents in all five formulations increased significantly after processing. Coliform count and Bacillus cereus counts were <10 cfu/g.

## 1. Introduction

Most Ghanaians look out for easily prepared foods for breakfast to suit their busy schedules [1]. They prefer processed breakfast cereals which have been designed to minimize the time and labour involved in breakfast preparation. Breakfast cereals are usually eaten as the first meal of the day [2] with grains as the main ingredient. Breakfast cereal can be eaten partially/shortly cooked while others are eaten cold or mixed with milk, yoghurt, or fruit juice.

The most common cereal used in the preparation of breakfast meals in Ghana is maize [3]. There are two common types of maize in Ghana: the white maize (*Obatanpa*) and yellow maize (*Abontem*) [4]. Yellow maize, however, is more nutritious due to its richness in carotenoids such as beta carotene and anthocyanins [5] and tastier compared to white maize [6]. Yellow maize is also rich in minerals, vitamins, and carotenoids, particularly beta carotene which is a precursor of retinol [6] as well as gelatinization, pasting, and crystallization functionalities which makes it useful in breakfast cereal production [7].

Coconut (*Cocos nucifera* L.) is a common fruit in Ghana which is normally eaten raw or used as additives in cooking and in the preparation of fruit juices, toffees, and drinks. Coconut is rich in minerals, flavonoids, and other phenolic compounds as stated by Carandang [8] which imparts sweet flavour to food, and the oil content soothes surfaces, thereby facilitating its processing.

The ready-to-eat breakfast cereals available in Ghana are mostly imported and thereby limiting variety because not every variety desired by Ghanaians can be imported into the country. None of these breakfast cereals seen on the Ghanaian markets, however, make use of coconut as a component. In addition, the imported breakfast cereals are expensive which most people cannot afford.

This research therefore seeks to include the use of coconut as a component with yellow maize in the production of a suitable ready-to-eat breakfast cereal and then assess consumer acceptability of the product. This research will also provide another variety and a local option (breakfast cereal) made with already abundant raw materials that can be readily available and affordable to consumers.

## 2. Methodology

### 2.1. Materials

Yellow maize sample was obtained from the Centre for Scientific and Industrial Research (CSIR), Crop Research Institute, located at Fumesua in Kumasi. The *Abontem* variety was selected and used based on its superior nutritional values, coupled with physicochemical properties such as swelling power over *Odomfo*, *Nwanwa*, *Honampa*, and *Golden jubilee* varieties.

A resistant hybrid of *Sri Lanka Green Dwarf* and *Vanuatu Tall variety* (SGD × VTT) samples of matured coconut [9] was also obtained from the CSIR-Oil Palm Research Institute in the Eastern Region.

### 2.2. Formulation of Product

Different formulations were obtained using the linear model and the mixture D-optimal model based on a preliminary study performed. Five different blends are obtained using Table 1.

### 2.3. Preparation and Processing of the Blends

Raw dried maize grains were steeped in deionised water for 12 hours, drained, and then milled into flour. Fresh coconut copra was washed in deionised water, chopped, and milled into paste using the Premier colloid mill (Surrey KE 1: 23 TZ, 84 ml with working clearance of 0.025). Mixtures obtained were made into dough using the ratio 6 : 1, that is, 6 kg of maize and coconut blend to one litre of water. Then, the 400 g of sugar, 50 g of salt, and 150 g of powdered milk were added afterwards. The dough was processed using a laboratory atmospheric double roller drum dryer (R. Simon's dryers, Nottingham, England, with WP of 7 bar and temperature of 169°C). The dough was fed into the drum dryer which dried it into flakes. The product was named *Zeaco flakes*.

### 2.4. Proximate Analysis

Proximate analysis was performed on the raw maize flour and the coconut paste using the Association of Official Analytical Chemists [10, 11] standard methods.

### 2.5. Microbial Analysis

All five samples were tested for total coliform count and Bacillus cereus counts using the pour plate technique and incubated at 25°C for 72 hours. The colonies were counted using the colony counter.

### 2.6. Sensory Evaluation

Sensory evaluation was carried out in a sensory laboratory in the Department of Food Science and Technology, KNUST. Consumer preference testing (acceptability testing) was performed by fifty untrained panellists. Sensory attribute of taste, aroma, colour, texture, and appearance and overall acceptability were assessed using a seven-point hedonic scale from 1 (dislike extremely) to 7 (like extremely). Panellists were also allowed to give extra comments.

Panellists were served with coded samples randomly and were provided with water and tasteless biscuit as palette cleansers in-between samples.

### 2.7. Statistical Analysis

The sensory analyses were performed, and the resulting values and responses were analysed statistically. The Design-Expert software was used in this analysis at *p* < 0.05. The statistical tool used was D-optimal design. The mean sensory data was analysed, and the graph followed a linear and cubic equations.

## 3. Results and Discussion

### 3.1. Proximate Analysis on Both Raw and Processed Cereal Formulations

The moisture contents in all the formulations reduced to a minimum (from 50 to 4.8, 49 to 4.53, 48 to 4.4, 47.8 to 4.15, and 45.4 to 4) after processing (Tables 2 and 3) which may limit microbial proliferation. With reference to Tables 2 and 3, a decrease in moisture content with reducing coconut percentage was observed. The coconut used was higher in moisture, and this therefore affected the formulations with higher coconut composition, raising their moisture contents in both raw and processed formulations. This implied that too much use of coconut used may result in high moisture food product, which may serve as a conducive environment for microbial growth.

**Figure 1:**
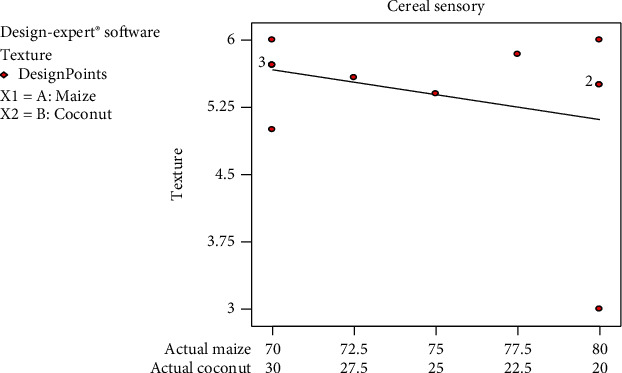
Mean graph for texture sensory responses on the samples.

Ash content can be estimated to represent overall mineral content in a food sample [12]. From Figure 1, the formulations recorded low mineral (ash) content in their raw state; however, it increased significantly after processing at *p* < 0.05. This confirmed the discovery by Mazaher et al. [13] that drum drying without precooking/pregelatinization increased the ash content. High mineral value in the processed cereal (Zeaco flakes) and the availability of antioxidants and micronutrients accounts for its ability to prevent several diseases and infections [14].

The protein content followed a significant increasing trend (at *p* < 0.05) with increasing yellow maize percentage except for raw formulations 75/25 and 77.5/22.5 whose difference was not significant. The processed cereals (Zeaco flakes) recorded a good quantity of proteins making it a good option for breakfast.

Fibre aids in digestion, preventing abrasion of the stomach walls and also soothes intestinal walls. Higher fibre contents were recorded with formulations with higher coconut percentage, which decreased significantly with decreasing coconut percentage. Coconut in its nature is a good source of roughages/fibre [15, 16] accounting for the increase in fibre content with increasing coconut quantity in the formulations. The high dietary fibre content in coconut is beneficial, as it serves as a functional food which is good for people of all ages [16].

Coconut naturally contains high amount of fat which most health-conscious people are concerned with. However, the fat in coconut is made up of unique medium chain fatty acids which according to Kabara [17] and Fernando et al. [18] are easily absorbed and metabolised by the liver.

In the research work of Mazaher et al. [13], the fat content of two drum dried products decreased significantly at *p* ≤ 0.05. This was contrary to the results in this study, where the fat contents of the produced cereal flakes increased significantly. This may be due to the difference in the raw materials used.

Maize is a good source of carbohydrate, and it was the main component of the breakfast cereal. Tables 2 and 3 shows that the carbohydrate in the cereal increased as the yellow maize in the formulation increased. The total energy in the cereals followed the same trend as that of the carbohydrate. It increased with increasing yellow maize content in the formulation. This was expected since maize is known to be high in carbohydrate content and therefore a high energy source [4, 19].

### 3.2. Sensory Responses from Panellists

An overall of nine runs comprising of five different formulations were made available using the Design-Expert's D-optimal design. These formulations were presented to panellist in batches to be analysed.

**Figure 2:**
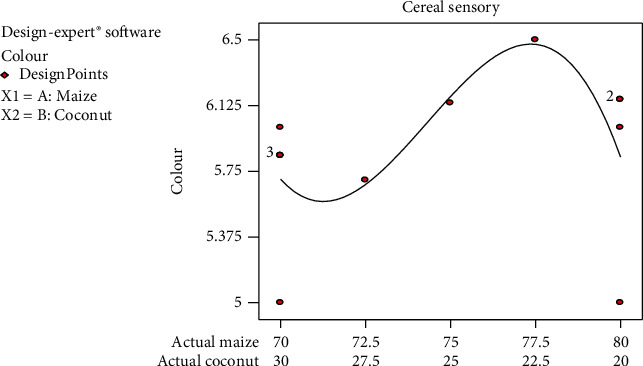
Mean graph for colour sensory responses on the samples.

The colour of the cereal was very appealing as confirmed from the mean graph for colour (Figure 2). This can be attributed to the carotenoid pigment in the maize. Panellists rated the colour between 5 (like moderately) and 7 (like extremely), indicating that, on the average, the panel liked *Zeaco flakes*' colour. However, the colour acceptability increased as the maize percentage increased to a peak at the 77.5/22.5 formulation and then declined with the 80/20 formulation. This may be because the increased intensity of the yellow colour of the cereal did not appeal to panellist. The colour acceptability rating was not significantly different from each other (*p* = 0.05). These findings are consistent with the colour acceptance of yellow maize porridge in a study by Govender et al. [20]. Yellow colour is appealing to the eye which made *Zeaco flakes* very attractive.

The texture was accepted by the panellist in all the various formulations since almost all the values were between 4 (neither like nor dislike) and 6 (like very much) but the acceptance slightly decreased with increasing maize composition. This could be attributed to the reduction of coconut copra content, which is high in fibre. The fibre aids in getting a crispy end product [21].

Panellists suggested that newly produced cereal would be more acceptable if the thickness was improved since *Zeaco flakes* was thinner in thickness and therefore hydrate faster when water was added, as compared to the existing Kellogg's or Crown field corn flakes on the market. Drum drying which was used to produce the yellow maize and coconut blend was designed to pick up only a thin layer of feed at a time to dry [22] and therefore produced thinner thickness of flakes. The flakes were also not as crispy as the other existing cornflakes on the market and break easily.

**Figure 3:**
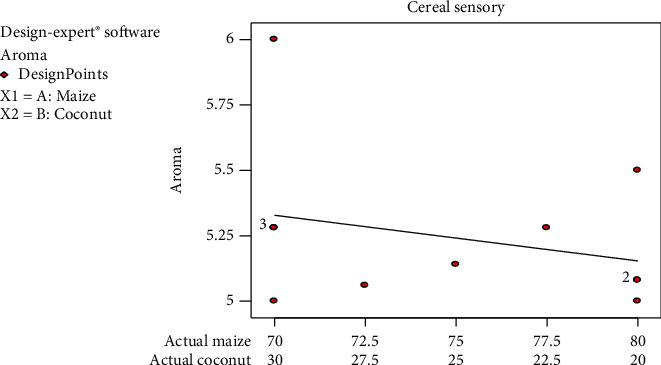
Mean graph for aroma sensory responses on the samples.

The aroma of *Zeaco flakes* followed a decreasing acceptability with increasing yellow maize composition. Panellists rated the aroma between 5 (like moderately) and 6 (like very much). The aroma of coconut which was imparted in *Zeaco flakes* was liked by the panel since Figure 3 shows that the panellists liked the aroma as the coconut percentage was increasing. Coconut has a distinct pleasant natural aroma which made the produced breakfast cereal smell better when compared with existing corn flakes on the market. From Figure 3, the mean score graph was between 5 and 6 which implied that panellists' assessment was in-between like moderately and like very much.

**Figure 4:**
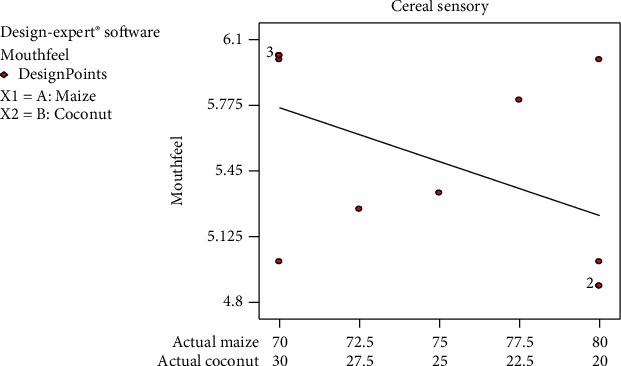
Mean graph for mouthfeel sensory responses on the samples.

Mouthfeel is an attribute which shows how panellist enjoyed the breakfast cereal while it is in the mouth. The mean linear graph for mouthfeel showed a decline with decreasing coconut composition in the cereal whereas the actual points on the graph showed an increasing trend with decreasing coconut composition. This implies that panellists liked the presence and the feel of coconut in their mouths and therefore disliked the product as the coconut percentage decreases. The mouthfeel values fell between 4 (neither like nor dislike) and 6 (like very much (Figure 4)) which indicates that the panellist enjoyed the produced breakfast cereal (*Zeaco flakes*). Coconut copra fibre may be the cause of good mouthfeel rated by panellists that could be why they liked the formulations with increasing coconut percentage.

**Figure 5:**
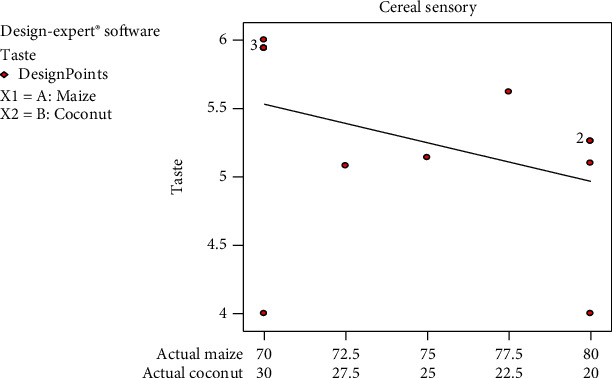
Mixture mean graph for taste sensory responses on the samples.

Yellow maize in its nature has a sweet taste, especially when it is harvested as sweet corn [6, 3], and the same applies to coconut, resulting in a very desirable taste in the breakfast cereal. The taste of *Zeaco flakes* mean score graph was between 4 and 5 which implied that panellists' assessment was in-between like moderately and neither like nor dislike (Figure 5).

**Figure 6:**
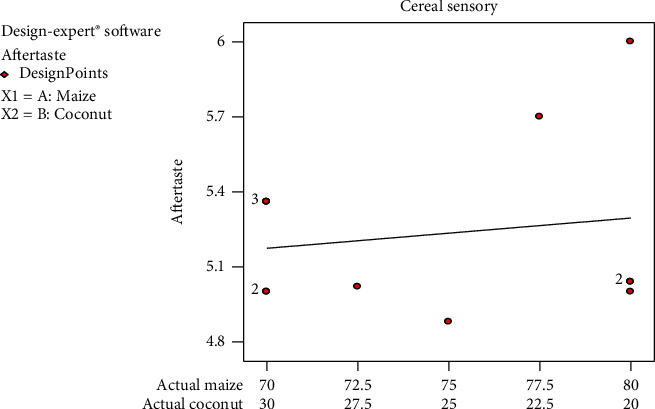
Mean graph for aftertaste sensory responses on the samples.

A desirable aftertaste was perceived by the panellists, implying that likeness increased as the yellow maize composition increased. However, the aftertaste likeness was not strong enough since its mean graph fell between 4 and 6, which means neither like nor dislike and like very much (Figure 6). This can be attributed to the sweet aftertaste of yellow maize due to the presence of sugar (fructose) [23]. Figure 6 shows that even though no undesirable aftertaste was perceived, panellists were not enthused by the product's aftertaste.

**Figure 7:**
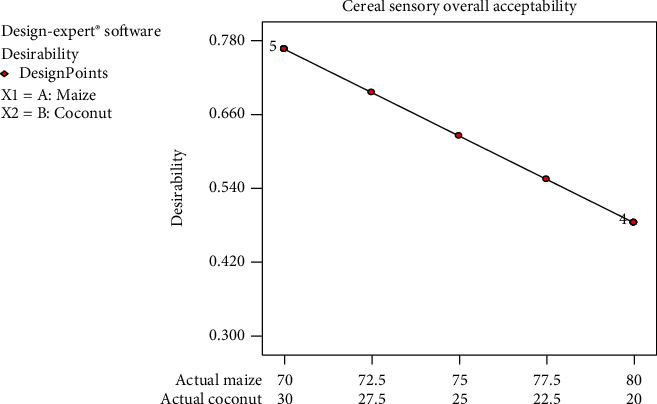
Mean graph for desirability (overall acceptability) of sensory responses on the samples.

Desirability is a combination of the results from all the sensory attributes, which is colour, texture, aroma, mouthfeel, taste, and aftertaste, and shows the overall acceptability of the product. From Figure 7, the mean score graph was between 4 and 5 which implied that panellists' assessment was in-between neither like nor dislike and like moderately. This evaluation could be due to the fact that *Zeaco flakes* is a new product and people are not used to it.

The desirability of the panellists for the samples followed a declining trend with increasing maize percentage. This is an indication that the formulation with the highest coconut content was desired most and the desirability decreased steadily as the coconut composition decreases in the formulation. The aroma, taste, and mouthfeel of all the cereal formulations decrease steadily as the coconut content decreases and falls within the ranges of 5.0–5.5. However, the cereal colour was liked more as the coconut percentage in the formulation decreases. All the responses from the sensory evaluation, when analysed, had no significant difference (*p* < 0.05), and none of them lacked fit. This means that all the values obtained from the sensory responses were within the limits set by the experimental design (D-optimal design). The taste of one formulation did not differ much from the other formulation (not significant at *p* < 0.05). Colour, aroma, texture, and aftertaste also showed no significant differences in-between the formulations. This may be due to the fact that the same food commodities were used to formulate all the five blends. The 77.5/22.5 formulation was preferred by panellist in terms of colour, taste, and aroma. However, in overall, the 70/30 formulation was mostly accepted in the entire sensory attributes.

### 3.3. Microbial Analysis

The resulting total coliform count in all the five formulations was <10 cfu/g, and Bacillus cereus count was <10 cfu/g. The microbial load in all the breakfast cereal formulations was counted as zero which implied that *Zeaco flakes* was safe for consumption.

## 4. Conclusion

Flaked breakfast cereal was produced using yellow maize and coconut, was nutritious, and was generally accepted by the sensory panellists.

Overall acceptability of *Zeaco flakes* rating was between 4 and 7 on the seven-point Hedonic scale, indicating that the panellist liked the product very much. There was no significant difference (*p* < 0.05) in the sensory responses despite the differences in component percentages. Coconut and yellow maize are therefore very suitable to be used as components/ingredients in the production of a convenient or ready-to-eat breakfast cereal. The produced ready-to-eat breakfast cereal is hygienic and safe for consumption.

## Figures and Tables

**Table 1:** Formulations for the ready-to-eat breakfast cereal.

Run	Coconut (%)	Yellow maize (%)	Sugar (g)	Salt (g)	Powdered milk (g)	Water (ml)
1	25.0	75.0	400	50	150	1000
2	30.0	70.0	400	50	150	1000
3	22.5	77.5	400	50	150	1000
4	20.0	80.0	400	50	150	1000
5	27.5	72.5	400	50	150	1000

**Table 2:** Proximate analysis outcome of the raw cereal blends (g/100 g).

Formulation	Moisture	Ash	Fat	Protein	Fibre	Carbohydrate	Energy
80/20	45.4	1.02	1	5.25	3.75	43.58	204.32
77.7/22.5	47.8	1.74	1.5	4.81	3.89	41.26	197.78
75/25	48	0.57	2.1	4.65	4.52	40.16	198.14
72.5/27.5	49	0.44	2	4.38	4.79	39.39	193.08
70/30	50	0.41	2.5	4.08	5.45	37.56	189.06

**Table 3:** Proximate analysis outcome on the processed cereal formulations (g/100 g) (Zeaco flakes).

Formulation	Moisture	Ash	Fat	Protein	Fibre	Carbohydrate	Energy
80/20	4.00	2.23	12.55	9.80	4.01	67.41	421.79
77.7/22.5	4.15	2.2	12.8	9.55	4.01	67.29	422.56
75/25	4.40	2.08	13	9.48	4.34	66.70	421.72
72.5/27.5	4.53	1.87	13.2	8.93	4.67	66.80	421.72
70/30	4.80	1.79	13.5	8.27	4.64	65	414.58

## Data Availability

The proximate analysis data and the sensory evaluation data used to support the findings of this study are included within the article and in the supplementary information file.

## Supplementary Materials

Supplementary Materials (I) Average of all the 50 sensory responses. (II) Microbial total viable count in *Zeaco flakes* after six months of storage. (III) Yeast and mould count in *Zeaco flakes* after six months.
