# Supplementary material for: Development and Sensory Assessment of Ready-to-Eat Breakfast Cereal
Source: Int J Food Sci. 2022 Aug 12;2022:4566482. doi: 10.1155/2022/4566482 (PMC9391194; doi:10.1155/2022/4566482)
Supplement: Supplementary Materials — (I) Average of all the 50 sensory responses. (II) Microbial total viable count in Zeaco flakes after six months of storage. (III) Yeast and mould count in Zeaco flakes after six months. [file 4566482.f1.docx]

AVERAGE OF ALL THE 50 SENSORY RESPONSES

| SAMPLES | COLOUR | TEXTURE | AROMA | MOUTHFEEL | TASTE | AFTERTASTE | OVERALL  ACCEPTABILITY |
| --- | --- | --- | --- | --- | --- | --- | --- |
| 80/20 | 6.16 | 5.5 | 5.08 | 4.88 | 5.26 | 5.04 | 5.5 |
| 77.5/22.5 | 6.5 | 5.84 | 5.28 | 5.8 | 5.62 | 5.7 | 5.92 |
| 75/25 | 6.14 | 5.4 | 5.14 | 5.34 | 5.14 | 4.88 | 5.44 |
| 72.5/27.5 | 5.7 | 5.58 | 5.06 | 5.26 | 5.08 | 5.02 | 5.42 |
| 70/30 | 5.84 | 5.72 | 5.28 | 6.02 | 5.94 | 5.36 | 5.76 |

Microbial total viable count in *Zeaco flakes* after six months of storage

| SAMPLE CODE | DILUTION | COUNT | MEAN COUNT/UNIT |
| --- | --- | --- | --- |
| 80/20 | 10^0^ | 13 | 12.5 x 10^1^ Cfu/g |
| 77.5/22.5 | 10^0^ | 2 | 4.0 x 10^1^ Cfu/g |
| 75/25 | 10^0^ | 2 | 1.0 x 10^1^ Cfu/g |
| 72.5/27.5 | 10^0^ | 46 | 44 x 10^1^ Cfu/g |
| 70/30 | 10^0^ | 1 | 0.5 x 10^1^ Cfu/g |

Yeast and mould count in *Zeaco flakes* after six months

| SAMPLE CODE | DILUTION | COUNT | MEAN COUNT/UNIT |
| --- | --- | --- | --- |
| 80/20 | 10^0^ | 0 | 0 |
|  | 10^0^ | 0 |  |
| 77.5/22.5 | 10^0^ | 1 | 1.0 X 10^0^ Cfu/g |
|  | 10^0^ | 1 |  |
| 75/25 | 10^0^ | 1 | 0.5 x 10^0^ Cfu/g |
|  | 10^0^ | 0 |  |
| 72.5/27.5 | 10^0^ | 0 | 0 |
|  | 10^0^ | 0 |  |
| 70/30 | 10^0^ | 0 | 0 |
|  | 10^0^ | 0 |  |
